# Laparoscopic sacrocolpopexy versus abdominal sacrocolpopexy for vaginal vault prolapse: long-term follow-up of a randomized controlled trial

**DOI:** 10.1007/s00192-022-05350-y

**Published:** 2022-09-16

**Authors:** Anique M. J. van Oudheusden, Josephine Eissing, Ivon M. Terink, Maarten D. H. Vink, Sander M. J. van Kuijk, Marlies Y. Bongers, Anne-Lotte W. M. Coolen

**Affiliations:** 1 Department of Gynecology and Obstetrics, Jeroen Bosch Hospital, P.O. Box 90153, 5200 ME ’s-Hertogenbosch, The Netherlands; 2 Department of Gynecology and Obstetrics, GROW, School for Oncology & Reproduction, Maastricht University, P.O. Box 616, 6200 MD Maastricht, The Netherlands; 3 Department of Gynecology and Obstetrics, Zuyderland Medical Centre, P.O. Box 5500, 6130 MB Sittard-Geleen, The Netherlands; 4 Utrecht General Practice Training Institute, University Medical Centre Utrecht, P.O. Box 85500, 3508 GA Utrecht, The Netherlands; 5 Department of Gynecology and Obstetrics, Isala Medical Centre, P.O. Box 10400, 8000 GK Zwolle, The Netherlands; 6 Department of Clinical Epidemiology and Medical Technology Assessment (KEMTA), Maastricht University Medical Centre +, P.O. Box 616, 6200 MD Maastricht, The Netherlands; 7 Department of Gynecology and Obstetrics, Máxima Medical Centre, P.O. Box 7777, 5500 MB Veldhoven, The Netherlands; 8 Department of Gynecology, Bergman Clinics, Marathon 1, 1213 PA Hilversum, The Netherlands

**Keywords:** Laparoscopic sacrocolpopexy, Abdominal sacrocolpopexy, Vaginal vault prolapse, Post-hysterectomy prolapse, Mesh exposure

## Abstract

**Introduction and hypothesis:**

The objective of this study was to evaluate long-term outcomes of laparoscopic sacrocolpopexy (LSC) versus abdominal sacrocolpopexy (ASC) for vaginal vault prolapse (VVP).

**Methods:**

Long-term follow-up of a multicenter randomized controlled trial (SALTO trial). A total of 74 women were randomly assigned to LSC (*n*=37) or ASC (*n*=37). Primary outcome was disease-specific quality of life, measured with validated questionnaires. Secondary outcomes included anatomical outcome, composite outcome of success, complications, and retreatment.

**Results:**

We analyzed 22 patients in the LSC group and 19 patients in the ASC group for long-term follow-up, with a median follow-up of 109 months (9.1 years). Disease-specific quality of life did not differ after long-term follow-up with median scores of 0.0 (LSC: IQR 0–17; ASC: IQR 0–0) on the “genital prolapse” domain of the Urogenital Distress Inventory in both groups (*p* = 0.175). Anatomical outcomes were the same for both groups on all points of the POP-Q. The composite outcome of success for the apical compartment is 78.6% (*n* = 11) in the LSC group and 84.6% (*n *= 11) in the ASC group (*p* = 0.686). Mesh exposures occurred in 2 patients (12.5%) in the LSC group and 1 patient (7.7%) in the ASC group. There were 5 surgical reinterventions in both groups (LSC: 22.7%; ASC: 26.3%, *p* = 0.729).

**Conclusions:**

At long-term follow-up no substantial differences in quality of life, anatomical results, complications, or reinterventions between LSC and ASC were observed. Therefore, the laparoscopic approach is preferable, considering the short-term advantages.

**Trial registration:**

Dutch Trial Register NTR6330, 18 January 2017, https://www.trialregister.nl/trial/5964

## Introduction

The prevalence of vaginal vault prolapse (VVP), requiring apical surgery, has been reported in 23% of women who underwent vaginal hysterectomy for pelvic organ prolapse (POP) [1]. The risk of developing VVP increases in the years after hysterectomy, especially in women whose initial hysterectomy was performed for POP [2, 3]. Pelvic floor symptoms due to POP can have a severe impact on women’s quality of life, requiring an effective treatment [4].

Sacrocolpopexy is one of the surgical options in the treatment of VVP, with success rates between 93 and 99% [5–8]. Sacrocolpopexy is associated with a lower risk of awareness of prolapse, recurrent prolapse on examination, repeat surgery for prolapse, and dyspareunia than other vaginal interventions for POP [9]. Previously, the results of the SALTO trial were published [10, 11]. In this multicenter RCT, we compared laparoscopic sacrocolpopexy (LSC) with abdominal sacrocolpopexy (ASC) as treatment for VVP, with a follow-up time of 12 months. The results showed less blood loss, a shorter hospital stay, and less related morbidity in favor of the laparoscopic group. There was a significant improvement in quality of life in both groups [10, 11].

Evidence for long-term clinical outcomes of LSC versus ASC is essential to reach consensus on the optimal surgical treatment, adequate patient selection and preoperative counselling. Therefore, this follow-up study was performed to evaluate the long-term outcome in terms of disease-specific quality of life of patients who participated in the SALTO trial.

## Materials and methods

### Study design

Details of the SALTO trial were published previously [10, 11]. In short, a multicenter randomized controlled trial was performed, comparing LSC and ASC as treatment for VVP, in four teaching hospitals and two university hospitals in the Netherlands. Eligible women with vault prolapse who met the inclusion criteria were informed about the trial, and randomized after consent. Inclusion criteria were women with a history of hysterectomy presenting with symptomatic vaginal vault prolapse, with or without concomitant cystocele or rectocele, who chose to undergo surgery.

This observational long-term follow-up study was approved by the ethical research committee (METC) of the Máxima Medical Centre (file number METC W17.015, CCMO NL60618.015.17) and by the board of directors of each of the participating hospitals, separately from the original SALTO trial. This trial was registered in the Dutch Trial Register (NTR6330). The study was developed and described in accordance with the Consolidated Standards of Reporting Trials (CONSORT) 2010 statement [12]. The results are reported by means of the joint International Urogynecological Association (IUGA)/International Continence Society (ICS) recommendations for reporting outcomes of surgical procedures for pelvic organ prolapse [13].

### Primary and secondary outcomes

The primary outcome of this trial was long-term disease-specific quality of life, measured with the Urogenital Distress Inventory (UDI). The primary outcome in our follow-up study is similar to the original SALTO trial. Secondary outcomes were the effects of the surgical treatment on POP-related functional symptoms such as micturition, defecation, sexuality, and patient satisfaction, using validated questionnaires. Moreover, long-term complications such as mesh exposure and retreatment were evaluated. Surgical retreatment was categorized according to the joint IUGA/ICS recommendations for reporting outcomes. Surgeries were subdivided into repeat surgery for the apical compartment, surgery for a different site (anterior or posterior compartment), surgery for complications, and surgery for non-POP-related conditions (e.g., stress urinary incontinence) [13, 14].

More outcome definitions were used in the literature after the initial SALTO study [14]. To make studies more comparable, we have added several secondary outcome measures. We analyzed composite outcome of success, defined as no POP beyond the hymen (apical compartment), absence of bothersome bulge symptoms, and no repeat surgery. Additionally, we examined surgical failure, which meant prolapse POP-Q ≥ stage 2 (in the apical compartment or in any compartment) or surgical reintervention. Last, anatomical failure (POP-Q ≥ stage 2) was evaluated [13–15].

### Data collection

All patients from the initial SALTO trial were sent a letter to ask for participation in this observational follow-up study. When they failed to respond, they were called and asked to participate. All participants gave new informed consent to participate in the long-term follow-up trial. They were asked to fill in various Dutch validated questionnaires and were invited to visit an outpatient clinic to undergo pelvic examination. The observer was an independent researcher, gynecologist or resident who had not performed the surgery and was trained in the POP-Q examination [16]. The observer was not blinded to the type of surgery, because of visible abdominal scars.

Disease-specific quality of life was tested with the UDI [17], the Defecatory Distress Inventory (DDI) [18], and the Incontinence Impact Questionnaire (IIQ) [17]. The UDI and DDI, containing of 19 and 11 items respectively, indicate whether complaints of micturition, prolapse, or defecation are present and to what extent these complaints are bothersome. These questionnaires consist of four-point Likert scales, ranging from “no bother” to “greatly bothersome.” The result of the IIQ questionnaire, composed of 13 questions, shows the disease-specific quality of life for urine incontinence. The score of each domain ranges from 0–100, a high score indicates more frequent or more bothersome symptoms (UDI and DDI), and hence, a poorer quality of life (IIQ). Patient satisfaction of their postoperative condition was verified by the Patient Global Impression of Improvement (PGI-I). The PGI-I is a seven-point Likert scale answering the question: “check the number that best describes what your post-operative condition is like now, compared with what it was like before you had the surgery” [19]. “Much better” or “very much better” was considered affirmative and presented as dichotomous outcome [9]. Furthermore, we evaluated sexual functioning using the Prolapse/Incontinence Sexual Questionnaire (PISQ), containing 12 questions. These items were scored on a five-point Likert scale ranging from 0 (always) to 4 (never), for which higher score indicates better sexual function [20, 21].

Bothersome bulge symptoms were measured using the UDI. A positive answer to any of the following questions is scored as a subjective recurrence: “Do you experience a sensation of bulging or protrusion from the vagina?” and “Do you have a bulge or something protruding that you can see in the vagina?”, in combination with a response “moderately bothersome” or “greatly bothersome” to the question “how much does this bother you?”

### Interventions

#### Laparoscopic sacrocolpopexy

Laparoscopic sacrocolpopexy was performed under general anesthesia using four trocars, one for the scope and three side trocars. The vaginal vault was elevated with a vaginal probe. The peritoneum from the promontory to the vault was incised laparoscopically by scissors to expose the rectovaginal and vesicovaginal fascia. A type 1 polypropylene mesh was used, which was cut into two pieces; 3 cm wide and approximately 15 cm long. One piece of the mesh was attached anteriorly and another as low as possible on the posterior vaginal wall, using non-absorbable multifilament sutures. The mesh was fixated to the anterior part of the vaginal vault with four stitches, and six stitches were used to fixate the mesh posteriorly. The mesh was attached to the sacral promontory using staples and was peritonealized [10].

#### Abdominal sacrocolpopexy

The ASC was performed by a laparotomy under general anesthesia, preferably using a Pfannenstiel incision. The essence of the procedure was the same as for the laparoscopic procedure. The peritoneum from the promontory to the vault was incised to expose the rectovaginal and vesicovaginal fascia, extending to the sacral promontory. One piece of type 1 polypropylene mesh was attached between the vagina and the bladder anteriorly, and another as far down the posterior vaginal wall as possible. The sutures, the size of the mesh and its fixation were the same as in the laparoscopic approach. The two meshes were sutured to each other, after which only the posterior mesh was fixed to the longitudinal vertebral ligament by staples or non-absorbable sutures, depending on the surgeon’s preference. The mesh was peritonealized. All centers used polypropylene meshes and the same sutures [10].

### Sample size

Sample size calculation was performed for the initial SALTO trial and 74 patients were included accordingly [10]. Loss to follow-up from the initial trial was taken into account and a response rate of 60% was estimated. A difference of 15 points between the two groups on the “genital prolapse” domain from the UDI was considered a clinically relevant difference. The standard deviation of the UDI score was 15.8 [22]. With an *α* level of 0.05 and a 60% response rate, the calculated power would be 83% and was considered to be adequate.

### Statistical analysis

The domain scores were calculated for the UDI, DDI, IIQ, PISQ, and PGI-I questionnaires. To examine differences between the two groups the independent-samples *t* test was used for continuous variables. The Mann–Whitney *U* test was used in the case of non-normally distributed variables. For dichotomous variables, Pearson’s Chi-squared test was used. The log-rank test was used for survival analysis of the time to surgical retreatment. Two-sided significance tests were used, and a *p* value of less than 0.05 was considered to be statistically significant. All statistical analyses were performed using IBM SPSS for Windows (version 25).

## Results

**Fig. 1:**
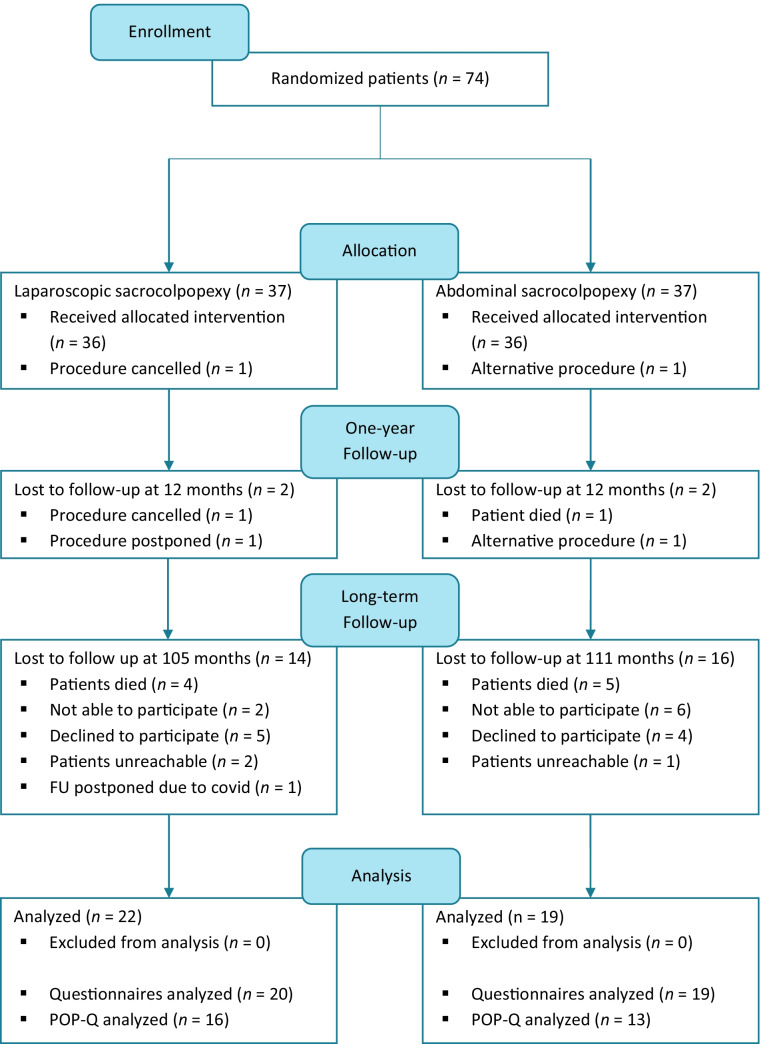
Flow diagram of study population. *FU* follow-up, *POP-Q* Pelvic Organ Prolapse Quantification

In the original trial 74 women were randomly assigned to LSC (*n* = 37) or ASC (*n* = 37) between 2007 and 2012. Figure 1 shows the flow diagram of the study population. In total 71 participants were eligible for long-term follow-up, 36 participants in the LSC group and 35 patients in the ASC group. We included 22 patients (61.1%) from the LSC group and 19 patients (54.3%) from the ASC group. Fourteen patients (38.9%) were lost to follow-up in the LSC group versus 16 patients (45.7%) in the ASC group; 9 patients died and 8 patients were not able to participate owing to old age or serious health conditions (unrelated to pelvic floor symptoms, e.g., terminal stage cancer). Nine patients were not willing to participate in this follow-up study. For most of them it was too much of a burden, none reported any POP-related complaints. In the LSC group 1 patient was lost to follow-up in the initial trial owing to postponed surgery, but agreed to participate now. Meanwhile, she received the allocated intervention (LSC).

Table 1 shows the baseline characteristics and peri-operative data of the patients in the SALTO trial. The median duration of follow-up was 109 months (9.1 years), 105 months (8.75 years) in the LSC group and 111 months (9.25 years) in the ASC group. In the LSC group 88.2% (*n* = 30) had two vaginal deliveries or more, compared with 94.1% (*n* = 32) in the ASC group. Also, the majority is postmenopausal at the time of surgery (LSC: 97.2%, *n* = 35; ASC: 100%, *n* = 37).

**Table 1:** Baseline characteristics and peri-operative data

	Laparoscopic sacrocolpopexy (*n* = 37)	Abdominal sacrocolpopexy (*n* = 37)	*p* value
Age at time of inclusion (years)			
Median (IQR)	65 (61–71)	67 (64–73)	N.A.
Parity			
No./total no. of patients (%)			N.A.
0	1/34 (2.9)	0/34 (0.0)	
1	3/34 (8.8)	2/34 (5.9)	
2	14/34 (41.2)	14/34 (41.2)	
3	13/34 (38.2)	9/34 (26.5)	
≥4	3/34 (8.8)	9/34 (26.5)	
Body mass index at time of inclusion (kg/m^2^)			
Mean (range)	25.3 (18–32)	25.9 (21–33)	N.A.
Menopausal status			
No./total no. of patients (%)			N.A.
Premenopausal	1/36 (2.8)	0/37 (0.0)	
Postmenopausal	35/36 (97.2)	37/37 (100.0)	
Urinary incontinence			
No./total no. of patients (%)			N.A.
None	20/35 (57.1)	15/35 (42.9)	
Stress	2/35 (5.7)	3/34 (8.8)	
Urgency	4/35 (11.4)	4/35 (11.4)	
Combined	9/35 (25.7)	13/35 (37.1)	
POP-Q stage apical compartment (point C)			
No./total no. of patients (%)			N.A.
Stage 0	0/32 (0)	1/34 (2.9)	
Stage 1	9/32 (28.1)	14/34 (41.2)	
Stage 2	9/32 (28.1)	9/34 (26.5)	
Stage 3	7/32 (21.9)	4/34 (11.8)	
Stage 4	7/32 (21.9)	6/34 (17.6)	
POP-Q stage 2–4			
No./total no. of patients (%)			N.A.
Anterior compartment prolapse			
(Ba ≥ −1)	24/30 (80)	21/32 (65.6)	
Posterior compartment prolapse			
(Bp ≥ −1)	10/28 (35.7)	20/32 (62.5)	
Follow-up duration (months)			
Median (IQR)	105 (87–126)	111 (79–117)	N.A.
Age at time of long-term follow-up (years)			
Median (IQR)	71 (68– 6)	76 (67–78)	0.549
Operative time (minutes)			
Median (IQR)	125 (108–135)	115 (94–129)	0.31
Estimated blood loss (ml)			
Median (IQR)	86 (10–100)	200 (100–300)	< 0.001
Hospital stay (days)			
Median (IQR)	2 (2–3)	4 (3–5)	< 0.001
Complications during surgery			
No./total no. of patients (%)	2/36 (5.6)	0/36 (0.0)	0.15
Bladder lesion (conversion)	1	0	
Bleeding (conversion)	1	0	
Complications during admission			
No./total no. of patients (%)	2/36 (5.6)	7/37 (18.9)	0.06
Fatal bowel perforation	0	1	
Wound dehiscence	0	2	
Pulmonary embolism	0	1	
Ileus	0	3	
Wound infection	1	0	
Pyelonephritis (re-admission)	1	0	

POP-Q stage 1: distal-most prolapse is > 1 cm above the hymen

POP-Q stage 2: distal-most prolapse is between 1 cm above and 1 cm beyond hymen

POP-Q stage 3: distal-most prolapse is > 1 cm beyond hymen, but no further than 2 cm less than total vaginal length

POP-Q stage 4: total prolapse

*POP-Q* pelvic organ prolapse quantification

The primary outcome of long-term disease-specific quality of life, measured with the UDI, was not different between groups. The median score for the domain “genital prolapse” was 0 (IQR 0–17) in the LSC group as well as in the ASC group (IQR 0–0; *p* = 0.175). On the other domains of the UDI, DDI, and IIQ, we did not observe any statistically significant differences, as is shown in Table 2. An improvement of “much better” or “very much better” on the PGI-I was reported by 11 patients (57.9%) in the LSC group, and 10 patients (58.8%) in the ASC group (*p* = 0.955). Sexual function was the same in both groups, with total PISQ scores of 34.2 (range 19–45) and 32.5 (range 28–37) in the LSC and ASC group respectively (*p* = 0.132). Thirty percent (*n* = 6) of the participants in the LSC group were sexually active, compared with 63% (*n* = 20) before surgery. In the ASC group there was also a reduction, from 45% (*n* = 14) to 10.5% (*n* = 2). Four patients were reported to have dyspareunia, two patients in each group (*p* = 0.102). Two patients also reported this pre-operatively, one in each group. From one patient, pre-operative data on sexuality are missing (ASC group) and the other patient was not sexually active before surgery (LSC group). Therefore, it was unclear whether the reported dyspareunia of these two patients occurred after surgery.

**Table 2:** Functional outcome and quality of life at long-term follow-up. Data are given in medians (IQR), unless stated otherwise

	Before surgery		Long-term follow-up		*p* value
	LSC (*n* = 34)	ASC (*n* = 31)	LSC (*n* = 20)	ASC (*n* = 19)	
Patient satisfaction (PGI-I)					
“Very much better” or “Much better”	N.A.	N.A.	11/19 (57.9)	10/17 (58.8)	0.955
Vaginal bulge symptoms					0.345
No bother*	3/29 (10.3)	3/30 (10)	14/20 (70)	18/19 (94.7)	
“Moderately bothersome” or “greatly bothersome”	26/29 (89.7)	25/30 (83.3)	3/20 (15)	0/19 (0)	
UDI					
Overactive bladder	33.3 (11–56)	44.4 (22–50)	16.7 (3–33)	22.2 (0–44)	0.762
Urinary incontinence	16.7 (0–50)	16.7 (0–42)	25.0 (0–33)	16.7 (0–42)	0.828
Obstructive micturition	0.0 (0–33)	16.7 (0–58)	0.0 (0–17)	0.0 (0–33)	0.901
Genital prolapse	66.7 (58–92)	66.7 (33–67)	0.0 (0–17)	0.0 (0–0)	0.175
Pain	16.7 (0–50)	33.3 (17–33)	0.0 (0–17)	16.7 (0–33)	0.061
DDI					
Obstipation	0.0 (0–17)	0.0 (0–33)	0.0 (0–17)	0.0 (0–17)	1.000
Obstructive defecation	4.2 (0–17)	8.3 (0–25)	0.0 (0–8)	8.3 (0–17)	0.531
Pain	0.0 (0–0)	0.0 (0–0)	0.0 (0–0)	0.0 (0–0)	0.749
Fecal incontinence	0.0 (0–17)	8.3 (0–33)	0.0 (0–29)	16.7 (0–33)	0.478
Flatus incontinence	33.3 (0–67)	33.3 (0–67)	16.7 (0–58)	0 (0–33)	0.396
IIQ					
Physical	25.0 (0–50)	0.0 (0–33)	0.0 (0–13)	0.0 (0–15)	0.897
Mobility	11.1 (0–33)	33.3 (11–44)	8.3 (0–23)	16.7 (8–42)	0.127
Social	11.1 (0–22)	11.1 (0–33)	0.0 (0–8)	0.0 (0–17)	0.967
Embarrassment	0.0 (0–17)	16.7 (0–17)	0.0 (0–13)	0.0 (0–13)	0.989
Emotional	11.1 (0–33)	22.2 (0–33)	0.0 (0–8)	8.3 (0–17)	0.322
Sexual function (PISQ)					
Sexually active	20/32 (62.5)	14/31 (45.1)	6/20 (30)	2/19 (10.5)	0.132
Dyspareunia	6/16 (37.5)	8/13 (61.5)	2/6 (33.3)	2/2 (100)	0.102
PISQ-12 total score, mean (range)	–	–	34.2 (19–45)	32.5 (28–37)	0.857
Behavioral-emotive, mean (range)	–	–	10.0 (6–15)	9.5 (9–10)	0.857
Physical, mean (range)	–	–	15.2 (4–20)	14.5 (11–18)	0.643
Partner-related, mean (range)	–	–	9.0 (6–10)	8.5 (8–9)	0.429

Values are given in median (interquartile range, IQR) or in number of participants/total number of participants (percentages), unless stated otherwise

Percentages were calculated using nonmissing data

PGI-I, patients who reported an improvement of “much better” or “very much better”

UDI and DDI; each item: 0 = no bothersome symptoms; 100 = most bothersome symptoms

IIQ; each item: 0 = best quality of life; 100 = worst quality of life

PISQ-12 total score: 0 = worst sexual function; 48 = best sexual function

PISQ-12 behavioral-emotive (items 1–4): 0 = worst function; 16 = best function

PISQ-12 physical (items 5–9): 0 = worst function; 20 = best function

PISQ-12 partner-related (items 10–12): 0 = worst function; 12 = best function

*LSC* laparoscopic sacrocolpopexy, *ASC* abdominal sacrocolpopexy, *PGI-I* Patient Global Impression of Improvement, *UDI* Urogenital Distress Inventory, *DDI* Defecatory Distress Inventory, *IIQ* Incontinence Impact Questionnaire, *PISQ* Prolapse/Incontinence Sexual Questionnaire

^a^Not all participants reported bothersome pelvic organ prolapse symptoms on the UDI questionnaire. They did so, however, at the outpatient clinic before inclusion in this trial

As shown in Table 3, the composite outcome of success for the apical compartment was 78.6% (*n* = 11) in the LSC group and 84.6% (*n* = 11) in the ASC group (*p* = 0.686). Surgical failure for the apical compartment was also statistically comparable, with 12.5% (*n* = 2) in the LSC group and 0% (*n* = 0) in the ASC group (*p* = 0.186). Anatomical failure and prolapse beyond the hymen also showed the same results for both groups (*p* = 0.186 and *p* = 0.359 respectively for the apical compartment).

**Table 3:** Outcome for pelvic organ prolapse (POP) after long-term follow-up

	Laparoscopic sacrocolpopexy (*n* = 16)	Abdominal sacrocolpopexy (*n* = 13)	*p* value
Composite outcome of success ^a^			
Apical compartment	11/14 (78.6)	11/13 (84.6)	0.686
Any compartment	7/14 (50)	10/13 (76.9)	0.148
Surgical failure ^b^			
Apical compartment	2/16 (12.5)	0/13 (0)	0.186
Any compartment	9/16 (56.3)	9/13 (69.2)	0.474
Anatomical failure ^c^			
Apical compartment (C ≥ −1)	2/16 (12.5)	0/13 (0)	0.186
Anterior compartment (Ba ≥ −1)	6/16 (37.5)	5/13 (38.5)	0.958
Posterior compartment (Bp ≥ −1)	6/16 (37.5)	6/13 (46.2)	0.638
Prolapse beyond hymen			
Apical compartment (point C > 0)	1/16 (6.3)	0/13 (0)	0.359
Anterior compartment (point Aa or Ba > 0)	3/16 (18.8)	1/13 (7.7)	0.390
Posterior compartment (point Ap or Bp > 0)	0/16 (0)	0/13 (0)	–
Reinterventions			
Surgical reintervention^d^	5/22 (22.7)	5/19 (26.3)	0.729
Time to surgical reintervention (months) mean (SEM)	41.2 (22.7)	55.8 (13.5)	0.814
Repeat surgery	0/22 (0)	0/19 (0)	
Surgery different site	3/22 (13.6)	4/19 (21.1)	
ACR	1	2	
PCR	2	2	
Surgery for complications	1/22 (4.5)	2/19 (5.2)	
Mesh removal	1	1	
Diagnostic laparoscopy	0	1	
Surgery for non-POP-related conditions	1/22 (4.5)	0/19 (0)	
MUS	1	0	
Pessary treatment	0/22 (0)	0/19 (0)	–
Physical therapy	3/21 (14.3)	3/19 (15.8)	0.894

All data are given in number of participants/total participants (percentages). Percentages were calculated using nonmissing data

POP-Q stage 1: most distal prolapse is > 1 cm above the hymen;

POP-Q stage 2: most distal prolapse is between 1 cm above and 1 cm beyond hymen;

POP-Q stage 3: most distal prolapse is > 1 cm beyond hymen, but no further than 2 cm less than total vaginal length;

POP-Q stage 4: total prolapse.

*POP-Q* pelvic organ prolapse quantification, *ACR* anterior colporrhaphy, *PCR* posterior colporrhaphy, *VSF* vaginal sacrospinous fixation, *SEM* standard error of the mean, *MUS* mid-urethral sling

^a^No POP beyond the hymen (in the apical compartment or any compartment), absence of bothersome bulge symptoms, and no surgical reintervention or pessary treatment.

^b^Prolapse POP-Q ≥ stage 2 (in apical compartment or in a any compartment) or repeat surgery or pessary treatment

^c^POP-Q ≥ stage 2

^d^One patient in the abdominal sacrocolpopexy group had surgery for complications and primary surgery for a different site

**Fig. 2:**
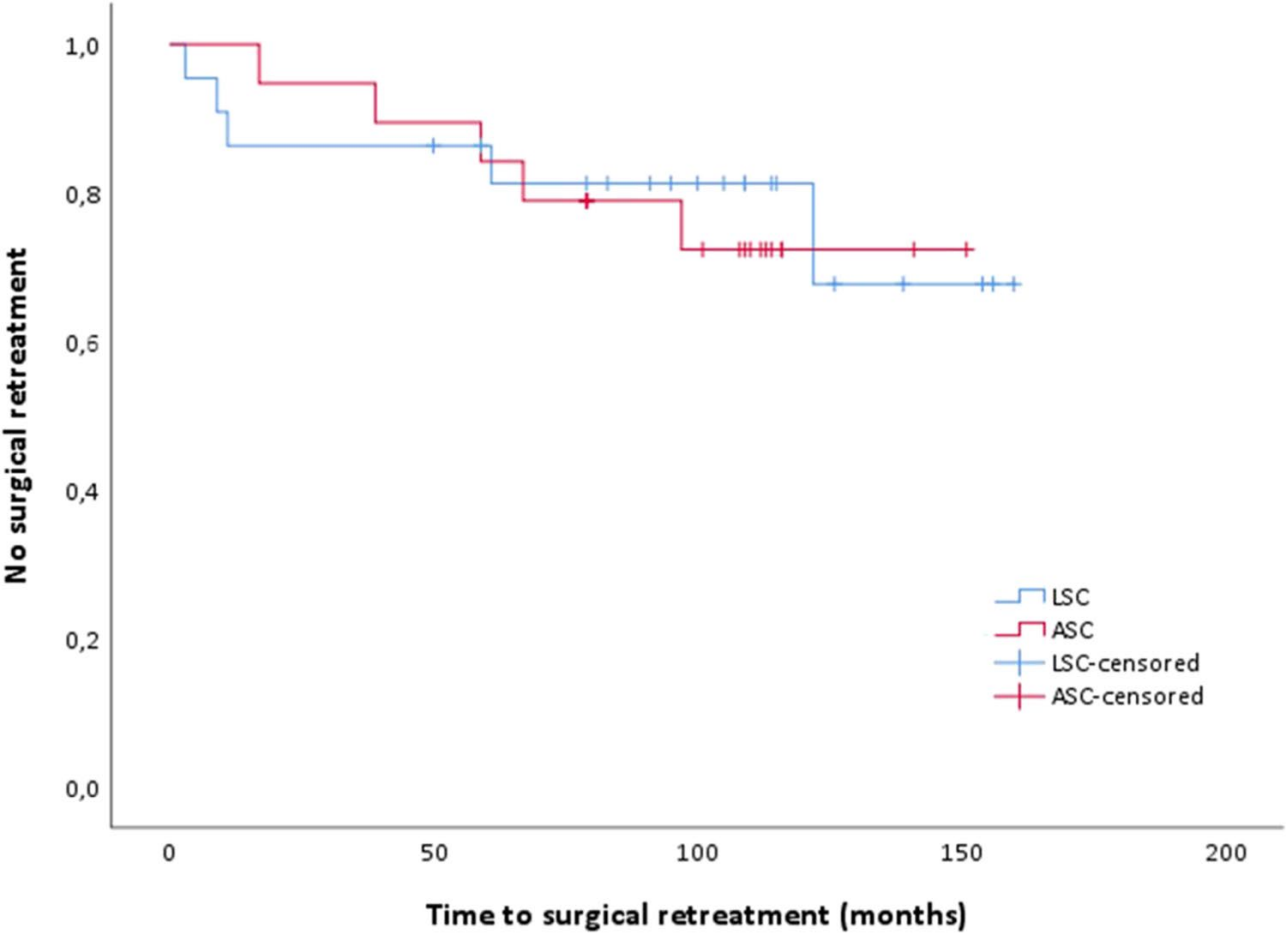
Survival analysis for time to surgical retreatment. Time (months) to surgical retreatment, *p* = 0.814. *ASC* abdominal sacrocolpopexy, *LSP* laparoscopic sacrocolpopexy

Last, Table 3 shows the reinterventions. In both groups five participants had surgical treatment, 22.7% in the LSC group and 26.3% in the ASC group (*p* = 0.729). Three patients in the LSC group and four patients in the ASC group underwent further surgery due to a bothersome cystocele or rectocele. One patient in the LSC group had de novo stress urine incontinence, for which she received a mid-urethral sling. Mean time to surgical reintervention (Fig. 2) was comparable in the two groups (LSC 41.2 months (SEM 22.7) versus ASC 55.8 months (SEM 13.5), *p* = 0.814). Two patients had surgery to remove the mesh, owing to severe complications. One patient presented with complaints of vaginal mesh exposure. The mesh got infected and extensive surgery was performed, 5.6 years (67 months) after she had undergone the ASC. During surgery it was discovered that the mesh fistulated through the vaginal vault. Adhesiolysis and resection of part of the ileum was performed. There was no descensus of the vaginal vault (POP-Q point C: −7) and an asymptomatic rectocele (POP-Q point Bp: 0) was left untreated. This surgery was otherwise uncomplicated and the patient made a good recovery. After 4 years, this patient had no POP-related complaints or pain. In the LSC group one patient also had a vaginal exposure and the mesh was infected. A robot-assisted procedure was performed to remove the mesh, 10.2 years (122 months) after she had undergone the LSC. The patient fully recovered from this complication. One patient from the ASC group had a diagnostic laparoscopy owing to complaints of abdominal pain. In each group, three patients were reported to have had pelvic floor physical therapy after the initial surgery (LSC 14.3% versus ASC 15.8%; *p* = 0.894). The initial sacrocolpopexy was without peri-operative complications for both patients.

Table 4 shows the mean POP-Q scores. Point C is at −4.7 in the LSC group (SD ± 3.9, range −8 to 8) and at −5.8 in the ASC group (SD ± 1.5, range −8 to −3), *p* = 0.353. The larger standard deviation is due to one patient in the LSC group, who had a stage 4 vaginal vault prolapse at long-term follow-up with “greatly bothersome” vaginal bulge symptoms.

**Table 4:** Pelvic Organ Prolapse Quantification (POP-Q) measurements

	1-year follow-up			Long-term follow-up		
	LSC (*n* = 29)	ASC (*n* = 29)	*p* value	LSC (*n* = 16)	ASC (*n* = 13)	*p* value
Aa	−1.3 ± 1.8 (−3 to 2)	−1.1 ± 1.6 (−3 to 3)	0.829	−1.4 ± 2.0 (−3 to 3)	−1.6 ± 1.5 (−3 to 2)	0.719
Ba	−1.3 ± 1.8 (−3 to 2)	−1.3 ± 1.3 (−5 to 8)	0.947	−1.3 ± 2.0 (−3 to 3)	−1.7 ± 1.3 (−3 to 1)	0.550
0C	−5.6 ± 2.3 (−8 to 0)	−5.1 ± 1.5 (−8 to −3)	0.621	−4.7 ± 3.9 (−8 to 8)	−5.8 ± 1.5 (−8 to −3)	0.353
GH	3.6 ± 0.7 (3 to 5)	4.0 ± 0.8 (3 to 5)	0.262	3.4 ± 1.0 (2 to 5)	3.6 ± 1.1 (1 to 5)	0.538
PB	3.1 ± 0.7 (2 to 4)	3.3 ± 0.7 (2 to 4)	0.624	3.0 ± 0.5 (2 to 4)	3.1 ± 0.6 (2 to 4)	0.723
TVL	7.8 ± 0.6 (7 to 9)	7.9 ± 1.6 (4 to 10)	0.896	7.7 ± 0.8 (6 to 9)	8.1 ± 1.4 (6 to 10)	0.394
Ap	−1.5 ± 1.3 (−3 to 0)	−1.6 ± 1.3 (−3 to 3)	0.840	−1.8 ± 1.2 (−3 to 0)	−1.8 ± 1.2 (−3 to 0)	0.924
Bp	−1.5 ± 1.3 (−3 to 0)	−1.6 ± 1.3 (−4 to 8)	0.840	−1.8 ± 1.2 (−3 to 0)	−1.7 ± 1.3 (−3 to 0)	0.571

POP-Q point Aa: located in the midline of the anterior vaginal wall 3 cm proximal to the external urethral meatus

POP-Q point Ba: the distal-most position of any part of the upper anterior vaginal wall from the vaginal cuff to point Aa

POP-Q point C: the distal-most edge of the vaginal cuff (hysterectomy scar)

POP-Q point GH (genital hiatus): measurement from the middle of the external urethral meatus to the posterior margin of the hymen

POP-Q point PB (perineal body): measurement from the posterior margin of the hymen to the mid-anal opening

POP-Q point TVL (total vaginal length): length of the vagina (centimeters) from the vaginal cuff to the hymen

POP-Q point Ap: located in the midline of the posterior vaginal wall 3 cm proximal to the hymen

POP-Q point Bp: the distal-most position of any part of the upper posterior vaginal wall from the vaginal cuff to point Ap

*ASC* abdominal sacrocolpopexy *LSC* laparoscopic sacrocolpopexy

Three mesh exposures and three suture exposures were described, and are shown in Table 5. Two mesh exposures, one in each group, were part of the complications described above. The other mesh exposure was in the LSC group and was left untreated, because it was only minor and without complaints. One patient in the LSC group and two patients in the ASC group had a suture exposure. The suture exposure for the patient in the LSC group was discovered at the follow-up visit for this study. She complained of vaginal blood loss and dyspareunia. After removal of this suture at the outpatient clinic she had no more complaints. In the ASC group, one suture exposure was discovered during an earlier visit of the patient to the outpatient clinic because of POP complaints, due to a rectocele. The suture was removed during subsequent vaginal surgery (posterior colporrhaphy). The suture exposure of the second patient in the ASC group was discovered by coincidence during vaginal examination for this follow-up study; the patient experienced no complaints and no treatment was performed.

**Table 5:** Complications after long-term follow-up

	Laparoscopic sacrocolpopexy (*n* = 16)	Abdominal sacrocolpopexy (*n* = 13)	*p* value
Complications	3/16 (18.8)	4/13 (30.8)	0.452
Mesh exposure with infection	1/16 (6.3)	1/13 (7.7)	
Mesh exposure	1/16 (6.3)	0/13 (0)	
Suture exposure	1/16 (6.3)	2/13 (15.4)	
Abdominal pain	0/16 (0)	1/13 (7.7)	

## Discussion

### Main findings

This observational long-term follow-up study of a multicenter randomized controlled trial shows that there was no difference in disease-specific quality of life whether after laparoscopic or after abdominal sacrocolpopexy, with median scores of 0.0 (LSC: IQR 0–17; ASC: IQR 0–0) on the “genital prolapse” domain of the UDI in both groups (*p* = 0.175). This corresponds with our previously published SALTO trial and LAS trial, both comparing the laparoscopic and the abdominal procedure, with 1-year follow-up [10, 23].

Composite outcome of success, surgical failure, and anatomical failure were the same in both groups for all compartments. We found no relation between the type of surgery and the compartment of the recurrence. Also, no relation was found between the pre-operative POP-Q stage and the compartment of the recurrence. Some patients had a recurrence in the same compartment as they did pre-operatively; others did not.

In our study, mesh exposures were reported in 12.5% and 7.7% in the LSC and ASC groups respectively. A retrospective cohort study from 2019 reports exposure rates of 1.4% [24]. We expect this to be an underestimation of the exposure rate, as they detected only patients with bothersome exposures. Three prospective cohort studies reported an exposure rate of 2.9%, 3.7%, and 4.5%. These studies had a shorter follow-up time, median of 60 months (5 years) instead of the 109 months (9.1 years) in our study, which could explain why they reported lower exposure rates [25–27]. Three suture exposures were found in our study population. In the SALTO trial nonresorbable sutures were used, which might contribute to these exposures. Nowadays, it is common practice to use resorbable sutures, which might lead to fewer suture exposures [28]. There were no other surgery-related risk factors in our study population, such as concomitant hysterectomy [29].

Patient satisfaction on the PGI-I is 57.9% (*n* = 11) in the LSC group and 58.8% (*n* = 10) in the ASC group (*p* = 0.955). This seems lower than patient satisfaction reported in other long-term follow-up studies [27, 30]. These studies report trials with a median follow-up time of 5 and 6 years, compared with the 9 years of our follow-up. The lower satisfaction in our trial might be due to a longer period of follow-up. It is understandable that patients find it more difficult to compare their situation now and before surgery, solely considering POP complaints after a longer period of time. The PGI-I asks patients to describe their post-operative condition, compared with how it was before surgery. Perhaps this question was not specific enough for the participants. Moreover, the PGI-I was only validated for a follow-up duration of 12 months [19].

### Strengths and limitations

We performed a randomized controlled trial, which is considered to yield the highest level of evidence when comparing two different treatment options. One of the main strengths of our study is the duration of follow-up, with a median of 109 months (9.1 years), which may be stated as “very long” (> 5 years) duration of follow-up, according to the IUGA/ICS joint report on the terminology for reporting outcomes of surgical procedures for pelvic organ prolapse [13]. To our knowledge, there is no other comparative study with similar or longer duration of follow-up for the laparoscopic versus the open abdominal approach to sacrocolpopexy. Another strength of our study is that we reported on additional outcomes; such as combined outcome measure, objective outcome, and subjective outcome [14, 15]. By conforming to more commonly used clinical outcomes, our data are easy to interpret and could be used for meta-analyses in the future.

One of the limitations of our study is the relatively high rate of loss to follow-up. However, the statistical power remains >80% for the primary outcome measure disease-specific quality of life. From the 36 eligible patients in the LSC group, 14 patients (38.9%) were lost to follow-up, compared with 16 (45.7%) of the 35 eligible participants in the ASC group. Nine patients died and eight patients were not able to participate owing to old age or serious health issues, which was beyond our control. Perhaps the SARS-CoV-2 pandemic added to the loss to follow-up; however, we have no complete data on this matter. Other studies reported attrition rates of 46% at 5 years [31], rising to 62% at 7 years [6]. Loss to follow-up generally increases with review time [9]. Although we opted for a higher response rate, our loss to follow-up is not more than could be expected.

Most of our study population were postmenopausal and multiparous, with two or more vaginal births. This makes our results mainly applicable for patients with comparable characteristics.

### Interpretation

The laparoscopic approach to sacrocolpopexy is preferable, compared with the open abdominal technique, mainly because of better short-term outcomes. The laparoscopic approach has less blood loss and a shorter hospital stay [10, 32]. Functional outcomes, complications, and retreatment were comparable for both techniques [10, 23, 32]. After a median follow-up of 109 months (9.1 years) the results are in line with the results after short-term follow-up. Therefore, laparoscopic sacrocolpopexy proves to be an effective and safe treatment for vaginal vault prolapse. More is known about patient-related and surgery-related risk factors for developing mesh exposure after sacrocolpopexy. Patients should be counseled accordingly and gynecologists should consider adjusting their technique to minimize the risk of mesh-related complications [29, 33]. LSC is a difficult procedure with a long learning curve; therefore, we believe this surgery should be performed by experienced surgeons and centralized care is preferable when the volumes are low.

## Conclusion

At long-term follow-up there was no substantial difference in disease-specific quality of life, anatomical results on the POP-Q, complications as mesh or suture erosions, and reinterventions between the LSC and the ASC groups. Therefore, the laparoscopic approach of sacrocolpopexy is preferable, considering the previously discovered advantages in the short term.
